# The impact of problematic social media use on subjective well-being among higher vocational college students: The chain mediating role of psychological distress and sleep disturbance

**DOI:** 10.1097/MD.0000000000042542

**Published:** 2025-06-13

**Authors:** Ming Li, Ahmad Zamri Khairani, Wenxuan Jiang

**Affiliations:** a School of International Education, Jiangsu Maritime Institute, Nanjing, China; b National Higher Education Research Institute, Universiti Sains Malaysia, Gelugor, Malaysia; c School of Teacher Education, Weifang Engineering Vocational College, Weifang, China.

**Keywords:** higher vocational college students, problematic social network use, psychological distress, sleep disturbance, subjective well-being

## Abstract

This study explores the impact of problematic social media use (PSMU) on the subjective well-being (SWB) of higher vocational college students, emphasizing the mediating roles of psychological distress (PD), and sleep disturbance (SD). A cross-sectional survey was conducted among 706 students from 4 higher vocational colleges in Jiangsu Province, China. Validated instruments were employed to measure PSMU, PD, SD, and SWB. Structural equation modeling was applied to examine the direct and indirect relationships among these variables, with particular attention to the mediating, and serial mediating effects of PD and SD. The analysis revealed 3 critical findings: (1) PSMU significantly and negatively influences the SWB of higher vocational college students; (2) PD and SD partially mediate the negative relationship between PSMU and SWB; and (3) PD and SD function as serial mediators, further amplifying the negative impact of PSMU on SWB. These findings contribute to the growing body of research on digital behavior and mental health by elucidating the mechanisms through which PSMU undermines well-being. The study offers practical insights for educators and policymakers aiming to develop interventions that mitigate PSMU’s negative effects and promote the overall well-being of higher vocational college students.

## 1. Introduction

Subjective well-being (SWB) refers to an individual’s overall assessment of life quality based on personal standards and experiences.^[1]^ It comprises 3 core components: life satisfaction, positive emotions, and negative emotions.^[2]^ The development of SWB during youth is particularly crucial, as it significantly contributes to mental health stability^[3]^ and academic success.^[4]^ Higher vocational college students constitute a distinct demographic within China’s higher education system. Unlike undergraduate students, they often come from diverse socio-economic backgrounds, emphasize hands-on learning and skill-oriented training, and encounter unique career pressures due to the practical focus of their education on job-readiness. These vocational students frequently face heightened academic stress, employment uncertainties, financial concerns, and social stigma with vocational education,^[5]^ making their psychological well-being an urgent area of concern. Consequently, exploring the factors influencing SWB in higher vocational college students has profound theoretical and practical implications.

The rapid expansion of internet use and the pervasive role of social media in daily life have significantly reshaped how higher vocational college students interact with the world. Social media has become an integral part of their routines, but this reliance has also brought challenges. Problematic social media use (PSMU) has emerged as a pressing concern, negatively impacting students’ SWB.^[6]^ PSMU refers to a form of behavioral addiction marked by excessive preoccupation with social media, compulsive usage patterns, and the disproportionate allocation of time and attention to these platforms, often leading to adverse consequences in multiple life domains.^[7]^ Research has consistently shown that PSMU undermines higher vocational college students’ SWB.^[8,9]^

Among the mechanisms linking PSMU to SWB, psychological distress (PD) is a critical factor. PD refers to a negative psychological state arising from exposure to stressful events or chronic stress and encompasses emotional experiences such as anxiety, depression, and tension.^[10]^ Studies have highlighted a strong negative relationship between PD and SWB, showing that individuals with higher levels of PD report reduced life satisfaction, fewer positive emotions, and more negative emotions.^[11]^

Another important factor is sleep disturbance (SD), which significantly impacts an individual’s sense of well-being. SD includes difficulties in falling asleep, maintaining sleep, or waking early, often accompanied by insomnia, vivid dreams, or frequent nighttime awakenings.^[12]^ Empirical evidence indicates a negative association between SD and SWB, with SD eroding individuals’ ability to experience life satisfaction and positive emotions.^[13,14]^

While previous studies have examined the roles of PSMU, PD, and SD in influencing SWB, few have explored the specific pathways through which PSMU affects SWB. This gap is particularly evident among higher vocational college students—a demographic that has received less research attention compared to university students in other higher education in China, despite their distinct educational and developmental contexts.

To address this gap, the present study aims to investigate the impact of PSMU on the SWB of higher vocational college students, focusing on the mediating roles of PD and SD. Specifically, this study examines the serial mediation effects of PD and SD to uncover the mechanisms underlying the relationship between PSMU and SWB.

This research contributes to the existing literature by providing a nuanced understanding of how PSMU affects higher vocational college students’ SWB. Furthermore, it offers actionable insights for educators and policymakers to design interventions that manage social media use, alleviate psychological distress, improve sleep quality, and ultimately promote students’ overall well-being among higher vocational college students.

## 2. Literature review and hypothesis development

### 2.1. Problematic social media use and subjective well-being

PSMU has been found to negatively predict students’ SWB.^[8,9]^ The Social Comparison Theory, proposed by Leon Festinger, remarks that individuals tend to evaluate their abilities and perspectives by comparing themselves with others.^[15]^ On social media, users often display idealized aspects of their lives, which can lead others to feel inadequate in comparison to their own circumstances and achievements. This sense of inadequacy can foster envy and dissatisfaction, ultimately affecting their SWB. A substantial body of research, both domestically and internationally, indicates that PSMU has a direct negative impact on SWB.

For instance, Brooks^[16]^ conducted a survey among 209 undergraduate students in the United States, revealing that social media use has the potential to undermine students’ personal happiness. In a longitudinal study by Raudsepp and Kais^[17]^ involving 397 adolescent girls, it was discovered that mitigating PSMU could prevent depressive symptoms, thereby enhancing the SWB of adolescents. Additionally, Boer et al,^[18]^ in a comprehensive survey of 154,981 adolescents across 29 countries, identified that those with problematic media use reported diminished well-being. Based on these findings, the following hypothesis is proposed.

H1: Problematic social media use has a negative impact on higher vocational college students’ subjective well-being.

### 2.2. The mediating role of psychological distress

PSMU has been shown to positively related to PD.^[19,20]^ Self-determination theory provides an insightful perspective for understanding this phenomenon, suggesting that PSMU may hinder the fulfillment of needs for autonomy, competence, and relatedness, potentially leading to anxiety, depression, and other forms of PD.^[21]^ Empirical research has also delineated a positive prediction of PD by PSMU. For instance, Thorisdottir et al,^[22]^ in a longitudinal study of 2097 adolescents, found that higher social media use was significantly associated with symptoms of depressed mood, social anxiety, and physical anxiety. Similarly, Chang et al^[23]^ reported that PSMU significantly increased anxiety and depression in a longitudinal survey of 645 university students. In a meta-analysis, Keles et al^[24]^ demonstrated that various aspects of social media use—time spent, activity, investment, and addiction—are consistently correlated with PD.

PD is known to exert a negative effect on SWB.^[25]^ Affective Events Theory posits that emotional states directly influence SWB; positive emotions enhance SWB, while negative emotions diminish it.^[26]^ PD, characterized by negative emotions like anxiety and depression, suppresses positive emotional experiences, thereby reducing overall SWB. For example, Piumatti et al,^[27]^ in a cross-sectional survey of 637 Serbian and 705 Italian university students, found that PD directly reduced students’ SWB, diminishing their experience of happiness. Additionally, research by Duong,^[28]^ utilizing an online-based cross-sectional survey of 1521 students from Vietnamese universities, discovered that PD significantly and negatively predicted life satisfaction. This indicates that PD not only reduces positive emotional experiences but also increases the proportion of negative emotions, leading to a lower overall evaluation of life quality.

These findings suggest that students with higher PSMU experience elevated PD, which in turn lowers their SWB. Thus, the following hypothesis is proposed:

H2: Psychological distress mediates the relationship between problematic social media use and subjective well-being among higher vocational college students.

### 2.3. The mediating role of sleep disturbance

It has been found that PSMU can influence SD.^[29]^ According to Technological Addiction Theory, when individuals develop a dependency on social media, compulsive usage behaviors may emerge, making it difficult to cease usage even when it is time to sleep, thereby disrupting normal sleep patterns.^[30]^ Empirical research has also demonstrated a positive relationship between PSMU and SD. Empirical research supports this connection: Alzhrani et al^[31]^ found that smartphone addiction was significantly negatively related to sleep quality in a study of 773 healthcare students and workers. Similarly, Vernon et al^[32]^ showed that problematic social networking directly led to sleep disruption in adolescents.

Sleep disturbance negatively impacts SWB. Richter,^[33]^ in a longitudinal study with American college students, demonstrated that sleep deprivation correlates with lower psychological well-being. Freitag et al,^[14]^ in a cross-sectional survey of 199 individuals, found that sleep disturbances were associated with decreased psychological well-being. Lima et al.^[34]^ further revealed that extreme sleep durations (both short and long) were linked to lower SWB in a survey of adults.

Psychological distress is a significant predictor of SD. Marino et al^[35]^ showed that PD, particularly depression and anxiety, leads to poor sleep quality. Han et al^[36]^ found that changes in anxiety and PD contribute to short-term sleep disturbances, while Wang et al,^[37]^ in a survey of 19,372 participants, confirmed that PD increases the prevalence of sleep problems. Based on this, the following hypotheses are proposed:

H3: Sleep disturbance mediates the relationship between problematic social media use and subjective well-being among higher vocational college students.

H4: Psychological distress and sleep disturbance jointly play a chain mediating role in the relationship between problematic social media use and subjective well-being among higher vocational college students.

**Figure 1.**
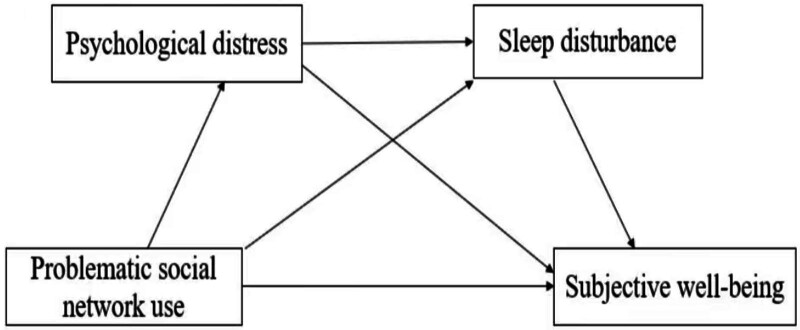
The research model.

Building on this above analysis, the study identifies the research gaps summarized in Table 1 and proposes a research model integrating problematic social media use, psychological distress, sleep disturbance, and subjective well-being, as illustrated in Figure 1.

**Table 1:** Research gaps identified in previous studies.

Research focus	Existing studies	Identified research gaps
PSMU and SWB	Previous studies confirm a negative relationship between PSMU and SWB (El Abiddine et al, 2022; Marttila et al, 2021; Brooks, 2015).	Limited research on higher vocational college students, who have distinct socio-academic stressors affecting their well-being.
Mediating role of psychological distress	Studies establish that PSMU is positively related to PD and that PD negatively affects SWB (Chen et al, 2020; Wu et al, 2016; Piumatti et al, 2019).	Few studies explore PD as a mediator in vocational students, particularly in China. The mechanism linking PSMU, PD, and SWB remains underexplored.
Mediating role of sleep disturbance	Research supports the link between PSMU and SD, as well as the negative impact of SD on SWB (Hussain and Griffiths, 2021; Vernon et al, 2018; Richter, 2015).	Insufficient evidence on SD as a mediator between PSMU and SWB, particularly in the context of higher vocational education.
Chain mediating role of PD and SD	Some studies suggest PD can lead to SD, further affecting SWB (Marino et al, 2022; Han et al, 2021).	The combined mediating effect of PD and SD in the relationship between PSMU and SWB has not been fully examined in higher vocational college students.

PD = psychological distress, PSMU = problematic social media use, SD = sleep disturbance, SWB = subjective well-being.

## 3. Methods

### 3.1. Study design

**Figure 2.**
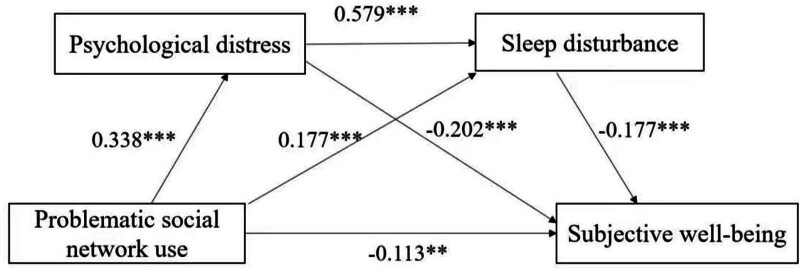
The path diagram.

This study employed a cross-sectional design to investigate the relationships between PSMU, PD, SD, and SWB among higher vocational college students. Data collection occurred between March 6, 2024, and May 15, 2024. Ethical approval was obtained from the Ethics Committee of Jiangsu Maritime Institute (approval number: 2024-0012), and the study was conducted in accordance with the ethical principles outlined in the Declaration of Helsinki (Fig. 2).

### 3.2. Setting

The study was conducted across 4 higher vocational institutions in Jiangsu province, China. These institutions were selected based on their diverse student demographics, providing a representative sample of higher vocational college students in the region. The recruitment and data collection processes were standardized across all institutions to ensure consistency. Data were collected using online questionnaires, which were distributed during scheduled class sessions through the Sojump software.

### 3.3. Participants

#### 3.3.1. Eligibility criteria

Students were eligible to participate if they were enrolled in one of the participating institutions, aged 18 years or older, and capable of providing informed consent. Students who reported medical or psychological conditions that might influence their responses were excluded.

#### 3.3.2. Sampling method

A stratified cluster sampling method was employed. A total of 800 questionnaires were distributed, and 706 were returned. After excluding questionnaires with logical inconsistencies, irregular completions, or identical responses to multiple consecutive items, 706 valid responses were retained, yielding an 88.25% response rate.

### 3.4. Variables

The study examined 4 primary variables:

PSMU: independent variable.

PD: mediating variable.

SD: mediating variable.

SWB: dependent variable.

### 3.5. Research instruments

#### 3.5.1. Problematic social media use scale

The PSMU was assessed using the revised Bergen Social Media Addiction Scale, as adapted by Lin et al^[38]^ from the original Bergen Facebook Addiction Scale.^[39]^ The Bergen Social Media Addiction Scale consists of 6 items, such as “I used social media in order to forget about personal problems,” scored on a 5-point Likert scale ranging from 1 (very rarely) to 5 (very often). The average of all item scores was calculated to provide a composite PSMU score, with higher scores indicating greater levels of problematic social media use. This scale has demonstrated strong psychometric properties and has been validated in previous studies.^[40]^ In the present study, the scale exhibited excellent reliability with a Cronbach α of 0.897.

#### 3.5.2. Psychological distress scale

PD was measured using the Kessler Psychological Distress Scale (K10) developed by Kessler et al.^[41]^ The K10 consists of 10 items, such as “Did you feel so nervous that nothing could calm you down?” Participants rated each item on a 5-point Likert scale ranging from 1 (strongly disagree) to 5 (strongly agree). The K10 has been widely validated and used in various populations, including university students, with proven reliability and validity.^[28]^ In this study, the scale demonstrated strong internal consistency, with a Cronbach α of 0.945.

#### 3.5.3. Sleep disturbance scale

SD was evaluated using the PROMIS 6-item sleep disturbance scale, as adopted by Full et al.^[42]^ This scale includes 6 items, such as “I had a problem with my sleep,” with 2 items assessing overall sleep quality and 4 items examining restfulness, difficulty falling asleep, and other sleep-related challenges over the past 7 days. Sleep quality was rated from 1 (very poor) to 5 (very good), while the remaining items were rated from 1 (not at all) to 5 (very much). Scores were reversed for positively worded items before calculating an overall average, with higher scores indicating greater sleep disturbance. This scale has demonstrated robust psychometric properties in prior research.^[28]^ In this study, the PROMIS 6-item sleep disturbance scale exhibited strong reliability, with a Cronbach α of 0.910.

#### 3.5.4. Subjective well-being scale

SWB was assessed using the 5-item Satisfaction With Life Scale developed by Diener et al.^[43]^ The Satisfaction With Life Scale assesses the cognitive dimension of SWB, with items such as “In most ways, my life is close to my ideal.” Responses were rated on a 7-point Likert scale from 1 (strongly disagree) to 7 (strongly agree). Higher scores indicated higher levels of life satisfaction and overall well-being. The scale has been widely used in research and has demonstrated strong reliability and validity.^[44]^ In this study, the scale exhibited excellent reliability, with a Cronbach α of 0.878.

### 3.6. Data sources/measurement

The data were collected through self-reported questionnaires administered during scheduled class sessions. To minimize response bias, participants were assured of the confidentiality and anonymity of their responses. The scales used in this study have been validated in diverse populations and exhibited robust psychometric properties in prior research.

### 3.7. Bias

To minimize potential biases and ensure ethical research practices, the study employed several measures:

1. Informed consent and voluntary participation: Prior to participation, students were provided with a detailed online informed consent form outlining the study’s purpose, procedures, potential risks, and confidentiality assurances. Only those who electronically signed the consent form and voluntarily agreed to participate were included in the study.
2. Screening for psychological conditions: To prevent potential distress, students who had been previously diagnosed with or were undergoing treatment for psychological disorders were excluded from participation. This exclusion was based on a self-reported screening question at the beginning of the questionnaire, where students were asked to indicate whether they had any diagnosed psychological conditions or were currently receiving treatment.
3. Use of validated instruments: The study utilized well-established scales with demonstrated reliability and validity to minimize measurement bias and ensure consistency in data collection.
4. Data quality control: Thorough data screening was conducted to remove incomplete, inconsistent, or logically implausible responses.

### 3.8. Study size

The sample size of 706 was determined based on the requirements for structural equation modeling, which suggests a minimum of 300 participants for stable model estimation.^[45]^ The final sample size exceeded this threshold, ensuring adequate statistical power.

### 3.9. Statistical analysis

Data analysis was conducted using SPSS 24.0 and Amos 24.0 software. The analyses included checking for common method bias, computing descriptive statistics, conducting correlation analyses, and assessing the reliability and validity of the scales. Mediation analyses were performed to examine the hypothesized relationships between problematic social media use, psychological distress, sleep disturbance, and subjective well-being. Indirect effects were tested using bootstrapping (1000 samples) to estimate confidence intervals.

## 4. Results

### 4.1. Participant flow

A total of 800 questionnaires were distributed to higher vocational college students across 4 institutions in Jiangsu Province. Of these, 706 were returned, resulting in a response rate of 88.25%. After excluding questionnaires with logical inconsistencies, irregular completions, or identical responses to consecutive items, all 706 were deemed valid and included in the analysis.

### 4.2. Participant characteristics

The participants included 55% males and 45% females; 28.3% were freshmen, 36.3% sophomores, and 35.4% juniors. Additionally, 55.5% of the students were from urban areas, while 44.5% were from rural regions. All variables of interest—PSMU, PD, SD, and SWB—were complete, with no missing data reported.

### 4.3. Common method bias test

To address the potential influence of common method bias, which can arise from factors such as the measurement environment, survey instructions, and contextual influences,^[46]^ Harman single-factor test was employed using SPSS 24.0. This analysis focused on key variables: PD, SD, and SWB. An unrotated principal component analysis identified 4 factors with eigenvalues >1, and the first factor explained 39.776% of the total variance—below the critical threshold of 40%. This result suggests that common method bias is not a concern for the current dataset, ensuring the validity of the subsequent findings.

### 4.4. Descriptive statistics and correlation analyses

Descriptive statistics, including the mean and standard deviation, were calculated for PSMU, PD, SD, and SWB. Additionally, Pearson correlation analysis was conducted to explore relationships among these variables. As shown in Table 2, several significant correlations were identified:

**Table 2:** Descriptive statistics and correlation analysis (N = 706).

	M	SD	1	2	3	4
Problematic social network use	2.605	0.851	1			
Psychological distress	2.451	0.823	0.338**	1		
Sleep disturbance	2.376	0.918	0.373**	0.639**	1	
Subjective well-being	4.057	1.098	‐0.247**	‐0.354**	‐0.348**	1

M = mean, SD = standard deviation.

** *P* < .01.

PSMU and SWB exhibited a significant negative relationship (*r* = −0.247, *P* < .01), indicating that higher levels of problematic social media use were associated with lower subjective well-being. PSMU and PD demonstrated a significant positive correlation (*r* = 0.338, *P* < .01), suggesting that increased social media use corresponded with elevated psychological distress. PD and SWB had a significant negative correlation (*r* = −0.354, *P* < .01), indicating that higher psychological distress was linked to lower levels of well-being. PSMU and SD showed a strong positive correlation (*r* = 0.639, *P* < .01), revealing that problematic social media use was associated with greater sleep disturbance. SD and SWB were significantly negatively correlated (*r* = −0.348, *P* < .01), suggesting that more severe sleep disturbances were related to reduced well-being. PD and SD were also positively correlated (*r* = 0.373, *P* < .01), indicating that higher levels of psychological distress were associated with more significant sleep disturbances.

Among these relationships, PSMU and SD had the strongest correlation (*r* = 0.639, *P* < .01), emphasizing the prominent role of sleep disturbances in the negative effects of problematic social media use. Conversely, the relationship between PSMU and SWB was weaker in comparison.

### 4.5. Testing for mediation effect

Structural equation modeling was used to assess the mediating effects of PD and SD in the relationship between PSMU and SWB. The analysis followed the bootstrap method proposed by MacKinnon,^[47]^ using 5000 bootstrap samples with a 95% confidence level for significance testing. The model fit indices were excellent, with the following values: 2/df = 1.403, IFI = 0.991, CFI = 0.991, TLI = 0.990, GFI = 0.969, AGFI = 0.957, and RMSEA = 0.021. All these values met the recommended thresholds (Zhang et al, 2020), indicating a strong model fit.

Mediation effects were considered significant if the 95% confidence intervals for the Bias-Corrected and Percentile methods did not include zero.^[47]^ The analysis was conducted using Amos 24.0, and the results are detailed in Table 3.

**Table 3:** Direct, indirect, and total effects of the hypothesized model.

Path relationship		Point estimate	Product of coefficient		Bootstrapping				
					Bias-corrected 95% CI			Percentile 95% CI	
			SE	*Z*-value	Lower	Upper	Lower		Upper
Test of indirect, direct, and total effects									
DistalIE	PSNU → PD → SD → SWB	‐0.046	0.016	‐2.875	‐0.087	‐0.024	‐0.077		‐0.023
FLEIE	PSNU → PD → SWB	‐0.078	0.030	‐2.600	‐0.131	‐0.030	‐0.131		‐0.028
PRIE	PSNU → SD → SWB	‐0.040	0.016	‐2.500	‐0.080	‐0.020	‐0.071		‐0.018
TIE	Total indirect effect	‐0.164	0.029	‐5.655	‐0.224	‐0.125	‐0.212		‐0.120
DE	PSNU → SWB	‐0.132	0.051	‐2.588	‐0.205	‐0.034	‐0.214		‐0.053
TE	Total effect	‐0.296	0.050	‐5.920	‐0.373	‐0.209	‐0.373		‐0.209
Percentage of indirect effects									
P1	DistalIE/TIE	0.283	0.089	3.180	0.158	0.469	0.146		0.447
P2	PDIE/TIE	0.475	0.159	2.987	0.191	0.709	0.198		0.710
P3	SDIE/TIE	0.242	0.088	2.750	0.118	0.429	0.111		0.404
P4	TIE/TE	0.554	0.125	4.432	0.392	0.817	0.381		0.787
P5	DE/TE	0.446	0.125	3.568	0.183	0.608	0.213		0.619

All indirect effects are statistically significant as the confidence intervals do not include zero.

CI = confidence interval, PD = psychological distress, PSMU = problematic social media use, SD = sleep disturbance, SWB = subjective well-being.

The direct effect of PSMU on SWB was significant (β = −0.132, *P* < .05), supporting Hypothesis 1. Additionally, both PD and SD were found to mediate the relationship between PSMU and SWB, with a total indirect effect of −0.164, further supporting Hypotheses 2–4. The indirect effects were decomposed into 3 pathways:

PSMU → PD → SWB: This pathway had an indirect effect of −0.078, with a 95% confidence interval of [−0.131, −0.030].

PSMU → PD → SD → SWB: This pathway produced an indirect effect of −0.046, with a 95% confidence interval of [−0.077, −0.024].

PSMU → SD → SWB: This pathway resulted in an indirect effect of −0.040, with a 95% confidence interval of [−0.080, −0.020].

For all 3 pathways, the bootstrap 95% confidence intervals did not include zero, indicating that the indirect effects were statistically significant. These findings provide compelling evidence that psychological distress and sleep disturbance play crucial roles in mediating the negative impact of problematic social media use on subjective well-being, supporting the proposed hypotheses.

## 5. Discussion

Empirical evidence from this study highlights the significant impact of PSMU, PD, and SD on the SWB of higher vocational college students. Despite existing research on the effects of PSMU, the specific pathways through which PSMU influences SWB via PD and SD remain underexplored. This study sought to address this gap by constructing a mediation model to investigate whether PSMU is indirectly related to vocational students’ SWB through PD and SD. The key findings and their implications are discussed below.

### 5.1. Problematic social media use negatively impacts higher vocational college students’ subjective well-being

The results confirm a significant negative relationship between PSMU and SWB, suggesting that excessive social media use is a robust predictor of reduced well-being among higher vocational college students. This aligns with previous studies.^[8,9,18]^ Two primary mechanisms may explain this relationship:

First, time displacement plays a critical role. Excessive social media engagement may cause students to neglect essential activities such as studying, physical exercise, socializing, and sleep. These missed opportunities can lead to deteriorating academic performance, declining physical and mental health, and weakened social skills, all of which diminish their overall well-being.

Second, social comparison is a contributing factor. Social media platforms often portray idealized versions of life, which can prompt users to make unfavorable comparisons between their reality and the perceived “perfect lives” of others. This phenomenon can foster feelings of envy, dissatisfaction, and unhappiness, further eroding students’ SWB. The findings strengthen the argument that PSMU is a substantial risk factor for poor well-being among higher vocational college students.

### 5.2. Psychological distress mediates the relationship between PSMU and SWB

The study reveals that PD partially mediates the relationship between PSMU and SWB, demonstrating that social media use not only directly impacts well-being but also indirectly diminishes it through heightened psychological distress. This aligns with prior research that links PSMU with elevated levels of anxiety, depression, and stress,^[22,23]^ and a concurrent reduction in SWB.^[27,28]^

One explanation for this mediation is that PSMU may be driven by external pressures or internal compulsions, which undermine feelings of autonomy, competence, and belonging. The constant exposure to social stimuli and the pressure to engage online can lead to chronic stress and mental exhaustion, triggering various forms of PD. In turn, these psychological burdens detract from students’ ability to maintain a positive emotional state, lowering their overall SWB. This finding highlights PD as a critical mediator in understanding how PSMU affects mental health and well-being.

### 5.3. Sleep disturbance mediates the relationship between PSMU and SWB

The study further identifies SD as a significant mediator in the relationship between PSMU and SWB. Consistent with previous findings,^[30,31]^ frequent use of social media has been shown to disrupt sleep patterns, leading to a cascade of negative effects on well-being. Sleep disturbances, often triggered by late-night social media use, excessive screen exposure, and irregular sleep schedules, can result in fatigue, irritability, and cognitive decline.^[14,34]^

These disruptions in sleep not only impair physical and mental health but also directly impact emotional regulation and life satisfaction. As sleep quality deteriorates, students are less able to manage stress and maintain a balanced emotional state, thereby further contributing to a decline in SWB. The mediation role of SD underscores the importance of healthy sleep hygiene in mitigating the harmful effects of PSMU on well-being.

### 5.4. The chain mediation of psychological distress and sleep disturbance

Finally, this study finds that PD and SD jointly mediate the relationship between PSMU and SWB in a chain-like manner. Regular exposure to social media can elevate psychological distress, which, in turn, disrupts sleep patterns. This compounded effect significantly amplifies the negative impact on students’ SWB. The interaction between PD and SD reflects a vicious cycle where heightened distress leads to poor sleep, which exacerbates psychological symptoms, further eroding well-being.

This finding highlights the interconnectedness of psychological and physiological health factors in shaping higher vocational students’ well-being. It suggests that interventions designed to address PSMU should not only target mental health (e.g., reducing anxiety and depression) but also promote healthy sleep practices to break the cycle of distress and sleep disruption. Recognizing the dual role of PD and SD provides a more comprehensive understanding of how social media behaviors can impact well-being, with practical implications for student support and mental health services in higher vocational education settings.

In summary, this study offers a nuanced perspective on the mechanisms through which PSMU affects higher vocational students’ well-being, emphasizing the mediating roles of psychological distress and sleep disturbance. These findings contribute to the growing body of literature on digital media’s impact on youth mental health and offer valuable insights for educators, policymakers, and mental health practitioners aiming to foster healthier social media habits and enhance the overall well-being of higher vocational students.

## 6. Implications and limitations

### 6.1. Implications

This study offers valuable contributions to both theoretical understanding and practical strategies aimed at improving SWB of higher vocational college students.

From a theoretical standpoint, this research enriches the corpus of existing scholarly work by underscoring the sequential intermediary function of PD and SD linking PSMU to SWB. It broadens our comprehension of the intricate dynamics at play within this connection and accentuates the necessity of examining a variety of elements when elucidating the behaviors that influence SWB among higher vocational college students. This study highlights the critical role of PD and SD as pivotal intermediaries in this nexus, casting light on the foundational processes that might be the focus of intervention and improvement initiatives.

From a practical perspective, this study offers valuable insights into designing effective strategies aimed at enhancing SWB among higher vocational college students. Interventions should concentrate not only on mitigating PSMU but also on diminishing PD and SD. To curb PSMU, educational institutions should contemplate the development of guidelines or policies that govern social media usage within the campus, establishing well-defined parameters for proper online behavior and offering support to students grappling with social media-related concerns. Furthermore, educators should guide students in developing healthy social media habits, empowering them to discern information on social media platforms and to think independently. They should also encourage higher vocational students to exercise their interpersonal skills offline, fostering real-life connections and preventing the paradox of being socially active online but isolated offline.

To alleviate PD, vocational colleges should establish psychological counseling centers staffed with professional counselors to provide students with accessible, specialized psychological services, thereby enhancing students’ self-awareness and emotional management skills. Additionally, teachers should create a supportive and inclusive classroom atmosphere, where students feel comfortable sharing their thoughts and feelings, can also help build resilience and self-confidence. To reduce SD, schools should initiate mental health education programs and activities to educate students on the significance of sleep and to provide methods for improving sleep quality. Students should establish good sleep hygiene, such as refraining from excessive use of electronic devices before bedtime to minimize the stimulation of blue light on the brain, and engaging in relaxing activities before sleep to help alleviate stress and unwind both mentally and physically.

### 6.2. Limitations

This study has several limitations that are worthy of consideration. First, the research design is cross-sectional, which limits the ability to infer causality between the variables. Future research could be conducted from longitudinal studies to further explore these relationships and evaluate the effectiveness of targeted interventions in mitigating the adverse effects of social media addiction. Additionally, the focus on higher vocational college students as the sole survey group may restrict the generalizability of the findings. Future research should aim to broaden the study population and increase the sample size by incorporating both longitudinal and cross-sectional methods. This would enable a more thorough exploration of the intrinsic causal relationships between PSMU, PD, SD, and SWB.

Second, while this study examined the mediating roles of PD and SD in the relationship between PSMU and SWB, other potentially influential factors, such as interpersonal relationships, academic performance, and coping strategies, were not addressed. Future studies should investigate these additional variables to provide a more comprehensive understanding of the determinants of SWB in vocational college students.

Third, although this study focused on the mediating effects of PD and SD, these are not the only factors influencing SWB. Future research should delve deeper into a broader range of psychological health factors, specific social media use patterns, and their interactions to better understand how these variables collectively impact the SWB of higher vocational college students. By expanding the scope of inquiry, subsequent studies can offer more nuanced insights into the complex relationships between PSMU and student well-being.

### 6.3. Generalizability of the study results

The generalizability of this study’s findings is subject to several considerations. First, the sample is drawn exclusively from higher vocational college students, limiting the direct application of results to other educational or demographic groups, such as university or higher school students. Second, conducted in higher vocational colleges in Jiangsu Province, China, the study’s findings are shaped by cultural and regional factors. Social norms around social media use, mental health attitudes, and sleep behaviors in this context may differ significantly from those in other regions or countries, limiting the transferability of results to diverse cultural or institutional settings.

## Author contributions

**Investigation:** Ming Li.

**Methodology:** Ming Li, Ahmad Zamri Khairani.

**Resources:** Wenxuan Jiang.

**Software:** Wenxuan Jiang.

**Supervision:** Ahmad Zamri Khairani.

**Validation:** Ming Li.

**Writing – original draft:** Ming Li.

**Writing – review & editing:** Ahmad Zamri Khairani.
